# Medico-Legal Society of Chicago

**Published:** 1896-04

**Authors:** 


### Medico-Legal Society of Chicago.

### THE HABITUAL CRIMINAL.

Report of the Special Committee on the papers of Dr. F. E. Daniel: "A Plea for Reform in Criminal Jurisprudence;" Maj. R. W. McClaughry:* "The Habitual Criminal;" Dr. Hamilton D. Wey:† "Sexual Perversity in a Reformatory, and its Treatment;" Mr. Matt. W. Pinkerton: "The Economical Treatment of the Habitual Criminal;" Mr. W. S. Elliott, Jr.: "The Legal Aspect of the Treatment of the Habitual and Sexual Criminal."

Mr. President and Members of the Medico-Legal Society:—Your Committee appointed to consider the papers and addresses presented at the last meeting of the Society on "The Habitual Criminal," after due consideration of these productions, and a review of the subject generally, beg leave to submit the following report:

1. That crime is increasing. A comparison of the census of 1850 with the census of 1890 shows that the population has increased 170 per cent, and criminals 445 per cent.

2. As causes of this increase in crime, we find vicious parentage, bad environments, intemperance, a constant increase in the urban population, unrestricted immigration, and the unreasonable manner in which laws are administered.

3. The Habitual Criminal is an abnormal man, this abnormality manifesting itself (1) physically by stigmata in cranial and cerebral development; by criminal physiognomy; by anomalies in the muscular, respiratory and circulatory systems; by anomalies in motor activity, and in physical sensibility; and (2) psychically, by moral insensibility; by a lack of forethought, by a low grade of intelligence; by vanity; by emotional instability, and by slang.

4. The proper treatment of the habitual criminal is a medico-legal question, and not a purely legal one. As a general proposition, the social and biological conditions of the prisoner, and not the accident of the crime, should determine the degree and kind of punishment.

For the diminution of crime we suggest that there should be rational modifications in the laws. (1) Marriage should be reg-

*In charge of Illinois State Reformatory.

†Surgeon in charge of Elmyra, N. Y., Reformatory.

ulated. The marriage license, in addition to the present requirements, should demand evidence that both the parties are in good health; that they are not alcoholic, nor narcotic inebriates; that they are not tuberculous, nor epileptic; that they have not active veneral disease; that they are not sexual perverts; that they are not insane, criminals, paupers. (2) The court should appoint suitable custodians for neglected children. (3) The pardoning power should be taken out of the hands of the governors of States and placed under the control of a board of pardons, who should be skilled in criminal anthropology. (4) In certain special cases asexualisation may be indicated, or, at least, is worthy of serious consideration. (5) There should be four classes of institutions in which criminals should undergo detention and treatment. (a) Local jails for the retention of prisoners awaiting trial in which segregation should be complete. (b) Reform schools for minor children. (c) Reformatories for elder criminals who can be reformed. (d) Penitentiaries for the lifelong incarceration of the incurables.

D. R. Brower. M. D.,

James Burry, M. D.,

G. Frank Lydston, M. D.

Committee.

